# Cellular energy stress induces AMPK-mediated regulation of glioblastoma cell proliferation by PIKE-A phosphorylation

**DOI:** 10.1038/s41419-019-1452-1

**Published:** 2019-03-04

**Authors:** Shuai Zhang, Hao Sheng, Xiaoya Zhang, Qi Qi, Chi Bun Chan, Leilei Li, Changliang Shan, Keqiang Ye

**Affiliations:** 1 Department of Medical Biochemistry and Molecular Biology, School of Medicine, Jinan University, 510632 Guangzhou, Guangdong China; 2 Department of Pathology and Laboratory Medicine, Emory University School of Medicine, Atlanta, GA 30322 USA; 3 The First Affiliated Hospital, Biomedical Translational Research Institute, Jinan University, 510632 Guangzhou, Guangdong China; 4 Department of Pharmacology and Emory Chemical Biology Discovery Center, Emory University, Atlanta, GA 30322 USA; 5 Department of Pharmacology, School of Medicine, Jinan University, 510632 Guangzhou, Guangdong China; 6 School of Biological Sciences, The University of Hong Kong, Hong Kong SAR, China

## Abstract

Phosphoinositide 3-kinase enhancer-activating Akt (PIKE-A), which associates with and potentiates Akt activity, is a pro-oncogenic factor that play vital role in cancer cell survival and growth. However, PIKE-A physiological functions under energy/nutrient deficiency are poorly understood. The AMP-activated protein kinase (AMPK) is an evolutionarily conserved serine/threonine kinase that is a principal regulator of energy homeostasis and has a critical role in metabolic disorders and cancers. In this present study, we show that cellular energy stress induces PIKE-A phosphorylation mediated by AMPK activation, thereby preventing its carcinogenic action. Moreover, AMPK directly phosphorylates PIKE-A Ser-351 and Ser-377, which become accessible for the interaction with 14-3-3β, and in turn stimulates nuclear translocation of PIKE-A. Nuclear PIKE-A associates with CDK4 and then disrupts CDK4-cyclinD1 complex and inhibits the Rb pathway, resulting in cancer cell cycle arrest. Our data uncover a molecular mechanism and functional significance of PIKE-A phosphorylation response to cellular energy status mediated by AMPK.

## Introduction

Phosphoinositide 3-kinase enhancer-activating Akt (PIKE-A) belongs to the PIKE family, a group of GTPases that interact with phosphoinositide 3-kinase (PI3K) and activate the PI3K/Akt pathway. PIKE-A is a proto-oncogene that has been reported to be upregulated in many cancers, including brain, breast, prostate, colon, ovary, liver, stomach, lung, cervix, and kidney, promoting glioblastoma cell proliferation and invasion^1–4^. Like variety of known proto-oncogenes, PIKE-A is usually through association with multitude binding partners, such as Akt, Unc-5 Netrin Receptor B (UNC5B), Focal Adhesion Kinase (FAK), cyclin-dependent kinase 5 (CDK5), NFκB, and STAT5a to interact with multiple signaling pathways to exercise function in cancer^5–10^. PIKE-A is localized in both the cytoplasm and nucleus and its cytoplasm–nucleus shuttling correlates with post-translational modification and physiological or pathophysiological functions. It is now clear that the phosphorylation of PIKE-A at S279 by CDK5 regulates nuclear translocation of PIKE-A and mediates growth factor-induced migration and invasion of human glioblastoma cells^8^. Our group previous studies showed that PIKE-A interacts with different partners, which mediated by Fyn phosphorylates on both its Y682 and Y774, then promoting cell survival and adipogenesis^9,11^.

The AMP-activated protein kinase (AMPK) is crucial cellular energy sensor that plays key role in adaptive responses to energy stress and energy homeostasis by promoting catabolic pathway of ATP production^12^. AMPK is activated by starvation or other stress (e.g., glucose deprivation, hypoxia, ischemia, and metabolic poisons treatment)^13^. Moreover, the adipokines leptin and adiponectin, cytokines such as interleukin-6 and ciliary neurotrophic factor, plant products such as berberine, resveratrol, and (−) epigallocatechin-3-gallate (EGCG), and small molecules such as metformin, minoimidazole-4-carboxymide-1-β-d-ribofuranoside (AICAR), thiozolidinedione (TZD), and A-769662 all can activate AMPK^14^. Upon activation, AMPK, as a heterotrimeric Ser/Thr kinase complex, phosphorylates its targets in order to stimulate catabolic processes dramatically, and at the same time to inhibit anabolic processes to restore cellular energy homeostasis, and chronically altering gene transcription and controlling cellular fate^12^. AMPK serves as a metabolic tumor suppressor that reprograms the cellular metabolism and triggers metabolic checkpoint on the cell cycle, which results in affecting cell proliferation, cell growth, cell survival, and autophagy through its actions on mTORC1, p53, and other modulators^15^. Recently, we provided new evidence supporting that the association between AMPK and PIKE-A was regulated by phosphorylation of PIKE-A mediated by Fyn, which is critical for inhibition of AMPK kinase activity, leading to cell proliferation arrest^3^. However, the precise molecular mechanisms of tumorgenesis driven by PIKE-A phosphorylation in the nucleus remain largely unknown.

14-3-3 proteins are highly expressed in human glioma U87 cells, while they cannot be detected in the normal human astrocyte SVGp12 cells^16^. They have gained a crucial position in cell biology owing to its involvement in many vital cellular processes, such as signal transduction, metabolism, transcription, apoptosis, protein trafficking, and cell cycle regulation^17,18^. However, in general, they regulate subcellular localization of target proteins, activity, or stability. This has raised the hypothesis that it is a crucial anchor protein in the cytoplasm to block its target proteins, which are imported into nucleus. There exist at least seven separate genes that encode seven 14-3-3 isoforms including β, γ, ε, ζ, σ, τ, and η in mammalian cells. However, different 14-3-3 isoforms may act as oncogenes or tumor suppressors in different types of cancers^19^.

Cyclin-dependent kinase (CDK) 4, a member of the cyclin-dependent kinase family, is important for cell cycle progression by promoting E2F transcription factor- and CDK2-dependent cell cycle progression, and promotes proliferation by inhibiting the retinoblastoma-associated protein (Rb1) tumor suppressor and by sequestering p27KIP1 and p21CIP1 (refs. ^20–22^). *CENTG1*, the gene encoding PIKE-A, co-amplified with CDK4 was observed 20 years ago; a recent report reveals that hsa-miR26a, CDK4, and PIKE-A comprise a functional integrated oncomir/oncogene DNA cluster, which promotes GBM tumorigenesis^23^. Qi et al.^21^ demonstrated that overexpression of PIKE-A or CDK4 alone in the TP53/PTEN double knockout GBM mouse model has longer latency of glioma onset and survival relative to co-express PIKE-A and CDK4 (ref. ^21^). These results reveal that PIKE coordinately acts with CDK4 amplification or overexpression to drive GBM tumorigenesis. We previously showed that PIKE-A inhibited AMPK by direct interaction which was mediated by the upstream tyrosine kinase Fyn. In parallel Fyn phosphorylates tumor suppressor LKB1. These events coordinately lead to hindering of the tumor suppressive actions of AMPK^24^.

In this report, we provide new evidence of a feedback regulation loop between AMPK and PIKE-A, showing that the phosphorylation status of PIKE-A mediated by AMPK is critical for its association with 14-3-3, and ultimately results in PIKE-A nuclear translocation. In the nucleus, the interaction between PIKE-A and CDK4 was enhanced by PIKE-A phosphorylation status, and resultedin the inhibition of CDK4 kinase activity, leading to the cell proliferation arrest. This discovery highlights a previously unappreciated regulation of PIKE-A by cellular energy status.

## Results

### AMPK phosphorylates PIKE-A on serine 351 and 377 residues

**Fig. 1:**
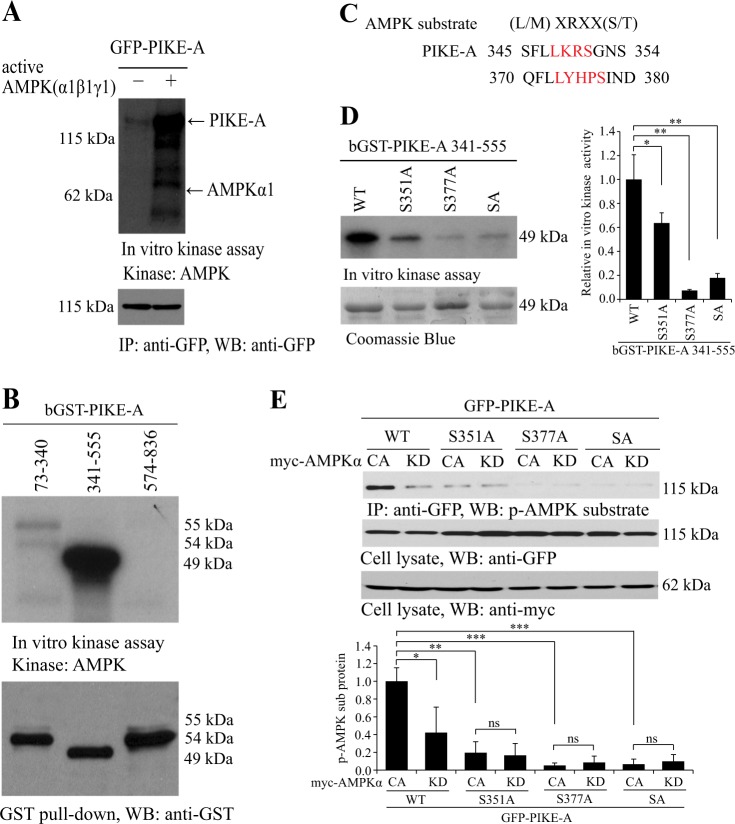
AMPK phosphorylates PIKE-A on serine 351 and 377 residues. **a** AMPK directly phosphorylates PIKE-A. Purified GFP-tagged PIKE-A recombinant protein was incubated with active AMPK (α1β1γ1). Phosphorylated proteins were detected by autoradiography. **b** A series of GST-tagged PIKE-A domain were incubated with active AMPK (α1β1γ1) and detected by autoradiography. **c** Serine 351 and 377 residues are potential phosphorylation sites of PIKE-A. These phosphorylation sites were colored in red and compared with the consensus AMPK substrates motif. **d** AMPK phosphorylates PIKE-A on serine 351 and 377 residues. Purified GST-tagged PIKE-A PH domain WT and mutants (S351A, S377A, and SA) were incubated with active AMPK (α1β1γ1). Phosphorylated proteins were detected by autoradiography. Quantification is shown at the right. **e** PIKE-A is a substrate of AMPK in vivo. HEK293 cells were co-transfected with GFP-PIKE-A WT or mutant (S351A, S377A, and SA), and either constitutively active AMPK (myc-AMPK T172D) or inactive AMPK (myc-AMPK T172A). PIKE-A was then precipitated and its phosphorylation was detected using an anti-phospho-(Ser/Thr) AMPK substrate antibody. Quantification is shown at the bottom. All results performed above are presented as mean ± SD from three independent experiments. *p < 0.05; **p < 0.01; ***p < 0.001; ns not significant

All cells coordinate cellular energy status with the change during cell growth, which is an energy-consuming process. AMPK directly phosphorylates key factors involved in multiple pathways to restore energy balance under energy stress^15,25^. We investigated whether PIKE-A could be phosphorylated by AMPK. Active AMPKα1β1γ1 strongly phosphorylated PIKE-A, as determined by in vitro phosphorylation assay (Fig. 1a). These results suggested that PIKE-A might be a substrate of AMPK. We then performed an in vitro phosphorylation assay using truncations and domains of PIKE-A and validated that PIKE-A phosphorylation sites are in PH domain (Fig. 1b and Figure S1A). When exploring the sequence of PIKE-A PH domain, we found that there are two optimal AMPK consensus substrate motifs around Ser-351/377 (Fig. 1c). Next, we test whether the S351/377 on PIKE-A are the phosphorylation sites. When S351/377 is mutated to Ala, the phosphorylation of PIKE-A by AMPK in vitro was completely abolished (Fig. 1d). To confirm this result, we examined the phosphorylation of PIKE-A using a pan-AMPK substrate antibody (Figure S1B). Indeed, the S351/377A mutant demonstrated no phosphorylation signal in cells transfected with active AMPKα (Fig. 1e). These results suggest that AMPK phosphorylates PIKE-A at Ser-351 and -377.

### AMPK phosphorylates PIKE-A and stimulates its nuclear translocation under cellular energy stress

**Fig. 2:**
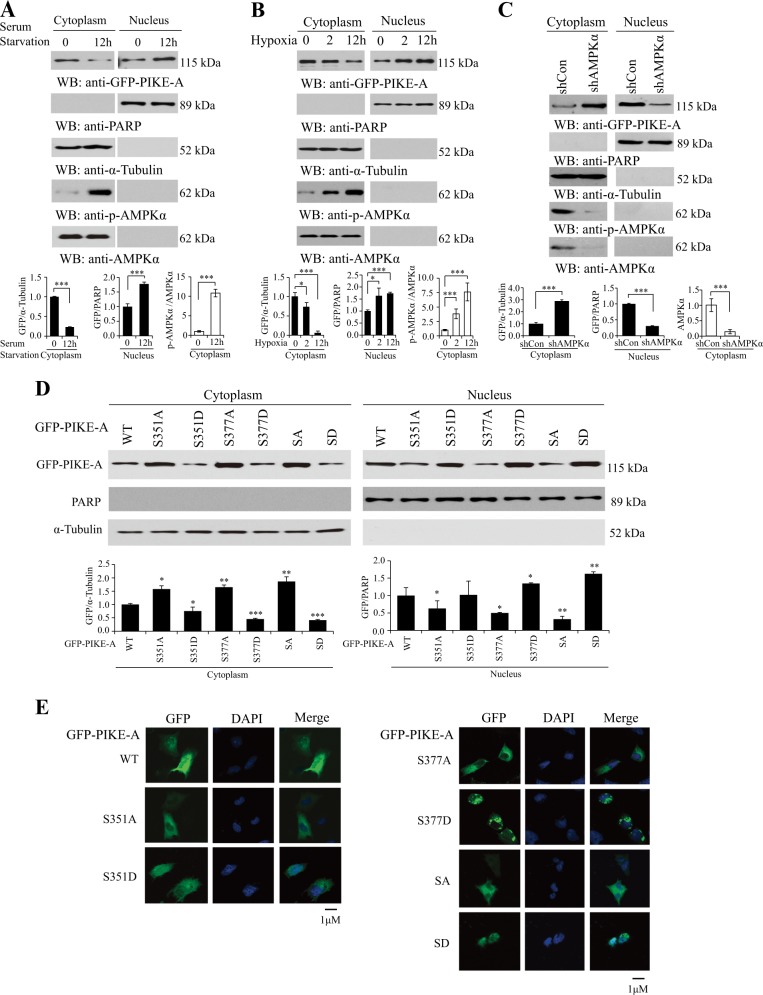
AMPK phosphorylates PIKE-A and stimulates its nuclear translocation. **a** Serum starvation stimulates PIKE-A nuclear localization. LN229 cells were transfected with GFP-PIKE-A WT and then serum starved for 12 h, followed by subcellular fractionation. Quantification is shown at the bottom. **b** Hypoxia enhances PIKE-A nuclear localization. HEK293 cells were transfected with GFP-PIKE-A and then hypoxia (1% O_2_) for 2 or 12 h, followed by subcellular fractionation. Quantification is shown at the bottom. **c** Knocking down AMPKα increases PIKE-A cytosolic localization and decreases PIKE-A nuclear localization. The subcellular fractionation was conducted in AMPKα shRNA or control vector cells transfected with LN229 cells. The purity of the cytosolic and nuclear fractions was confirmed by the absence of α-tubulin in the nuclear fraction and PARP in the cytosolic fraction. Quantification is shown at the bottom. **d** PIKE-A phosphorylation regulates its subcellular distribution. LN229 cells were transfected with GFP-PIKE-A WT or mutants (S351A, S351D, S377A, S377D, SA, and SD), followed by subcellular fractionation. The purity of the cytosolic and nuclear fractions was confirmed by the absence of α-tubulin in the nuclear fraction and PARP in the cytosolic fraction. Quantification is shown at the bottom. **e** PIKE-A phosphorylation regulates its subcellular distribution. LN229 cells were transfected with GFP-PIKE-A WT or mutants (S351A, S351D, S377A, S377D, SA, and SD) and then fixed and subjected to confocal imaging for the localization of PIKE-A and DAPI labeling for nuclei identification. All results performed above are presented as mean ± SD from three independent experiments. *p < 0.05; **p < 0.01; ***p < 0.001

As expected, serum starvation or hypoxia increased phosphorylation of AMPKα, in turn activated AMPK, which directly phosphorylates the PH domain of PIKE-A (Figure S1A and S1B). Previous studies show that PIKE-A is localized both in the cytoplasm and nucleus^8,26^. To explore the physiological consequence of PIKE-A phosphorylation of AMPK, we first monitored PIKE-A subcellular localization in LN229 cells under serum starvation or hypoxia condition by cytoplasmic and nuclear fractionation. The results showed that PIKE-A was translocated into the nucleus under energy stress (Fig. 2a, b). We blotted PARP and tubulin as the nuclear and cytoplasmic marker, respectively, showing minimum cross-contamination between these fractions.

AMPK directly monitors the cellular ATP/AMP ratio and regulates cell metabolism and growth in response to cellular energy status^15^. Therefore, we explored the role of AMPK on PIKE-A subcellular distribution. Notably, knocking down AMPK abolished nuclear PIKE-A (Fig. 2c). We then examined the PIKE-A subcellular localization in LN229 cells in the presence or absence of numerous small molecules which can activate AMPK. PIKE-A was usually localized in the cytoplasm, but translocated into the nucleus when treated with AICAR, Metformin, A23187, or H_2_O_2_, and at the same time increased phosphorylation of Thr 172 in AMPKα (Figure S2C).

To corroborate these findings, we next performed mutational analysis and generated diverse serine phospho-deficient S/A mutants and serine phospho-mimetic S/D mutants to determine the subcellular localization of PIKE-A. We found that substitution of S351 or S377 alone or together with D resulted in increased PIKE-A nuclear translocation (Fig. 2d). Similarly, immunofluorescence staining results revealed that PIKE-A S351A, S377A, and S351/377A mutant were largely present in the cytoplasm. However, PIKE-A S351D, S377D, and S351/377D mutants were predominantly present in the nucleus (Fig. 2e), suggesting that the phosphorylation of PIKE-A mediated by AMPK leads to accumulation of phospho PIKE-A in the nucleus. These data indicate that PIKE-A nuclear translocation correlated well with increased PIKE-A phosphorylation in an AMPK-dependent manner.

### 14-3-3β interacts phosphorylated PIKE-A by AMPK and stimulates its nuclear translocation

**Fig. 3:**
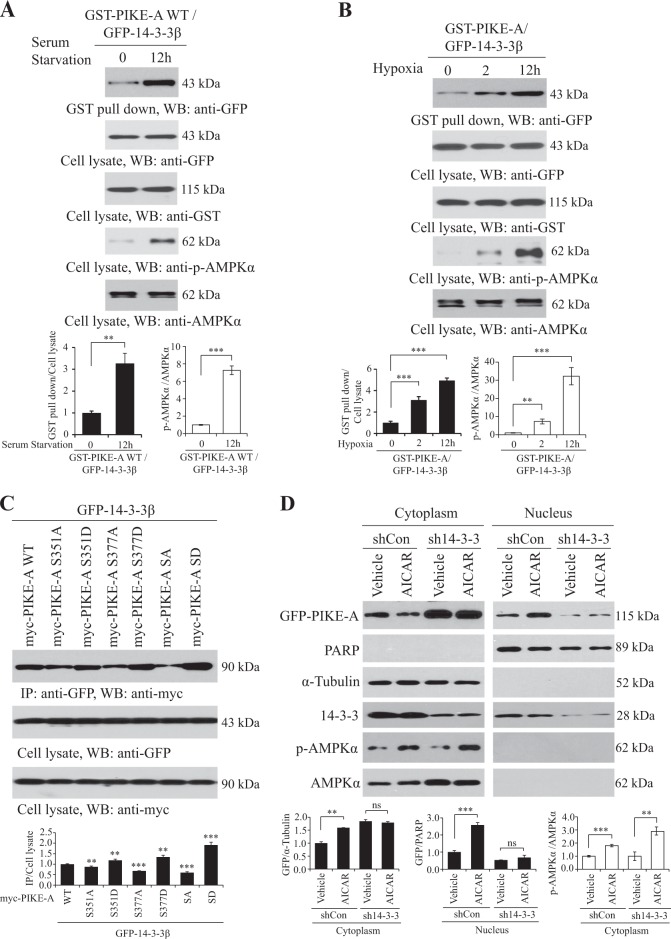
PIKE-A phosphorylation regulates its association with 14-3-3β. **a** Serum starvation enhances the interaction of PIKE-A and 14-3-3β. HEK293 cells were transfected with GST-PIKE-A and GFP-14-3-3β and then serum starved for 12 h, followed by immunoprecipitation. Quantification is shown at the bottom. **b** Hypoxia enhances the interaction of PIKE-A and 14-3-3β. HEK293 cells were transfected with GST-PIKE-A and GFP-14-3-3β and then hypoxia (1% O_2_) for 2 or 12 h, followed by immunoprecipitation. Quantification is shown at the bottom. **c** The myc-PIKE-A WT or mutants (S351A, S351D, S377A, S377D, SA, and SD) were co-transfected with GFP-14-3-3β into HEK293 cells. GFP-14-3-3β was immunoprecipitated and the co-precipitated proteins were analyzed using an anti-myc antibody. Quantification is shown at the bottom. **d** 14-3-3β regulates AMPK-phosphorylated PIKE-A nuclear translocation. 14-3-3β shRNA or control vector-transfected LN229 cells was treated with AICAR or not, followed by subcellular fractionation. The purity of the cytosolic and nuclear fractions was confirmed by the absence of α-tubulin in the nuclear fraction and PARP in the cytosolic fraction. Quantification is shown at the bottom. All results performed above are presented as mean ± SD from three independent experiments. **p < 0.01; ***p < 0.001, ns not significant

Through phosphoserine/threonine recognition motif, 14-3-3 proteins act as anchor proteins that play important roles in many regulatory processes, including intracellular protein targeting and stimulates its translocation^27,28^. To explore whether PIKE-A interacts with 14-3-3β proteins, we conducted a co-immunoprecipitation assay and found that PIKE-A physically associated with 14-3-3β and serum starvation or hypoxia stimulation enhanced this interaction (Fig. 3a, b). Similarly, in the presence of AICAR, Metformin, A23187, or H_2_O_2_ to stimulate PIKE-A phosphorylation, the binding between PIKE-A and 14-3-3β was increased (Figure S3A). These data suggest that the association between PIKE-A and 14-3-3β was mediated by AMPK phosphorylation.

To further confirm that the serine phosphorylation status on PIKE-A tightly correlated with the interaction between PIKE-A and 14-3-3β, we next performed a binding assay using PIKE-A mutants and 14-3-3β. We found that phospho-mimetic PIKE-A mutant (S351/377D, SD) increased the interaction slightly, while phospho-deficient PIKE-A mutant (S351/377A, SA) resulted in decreased interaction compare to PIKE-A WT (Fig. 3c). Noticeably, the depletion of 14-3-3β abolished PIKE-A nuclear translocation which escalated by AICAR, indicating that 14-3-3β is indispensable for AMPK-dependent PIKE-A nuclear translocation (Fig. 3d). Together, our data suggest that 14-3-3β interacts AMPK-phosphorylated PIKE-A and stimulates its nuclear translocation.

### AMPK-mediated PIKE-A phosphorylation stimulates its association with CDK4 in nucleus

**Fig. 4:**
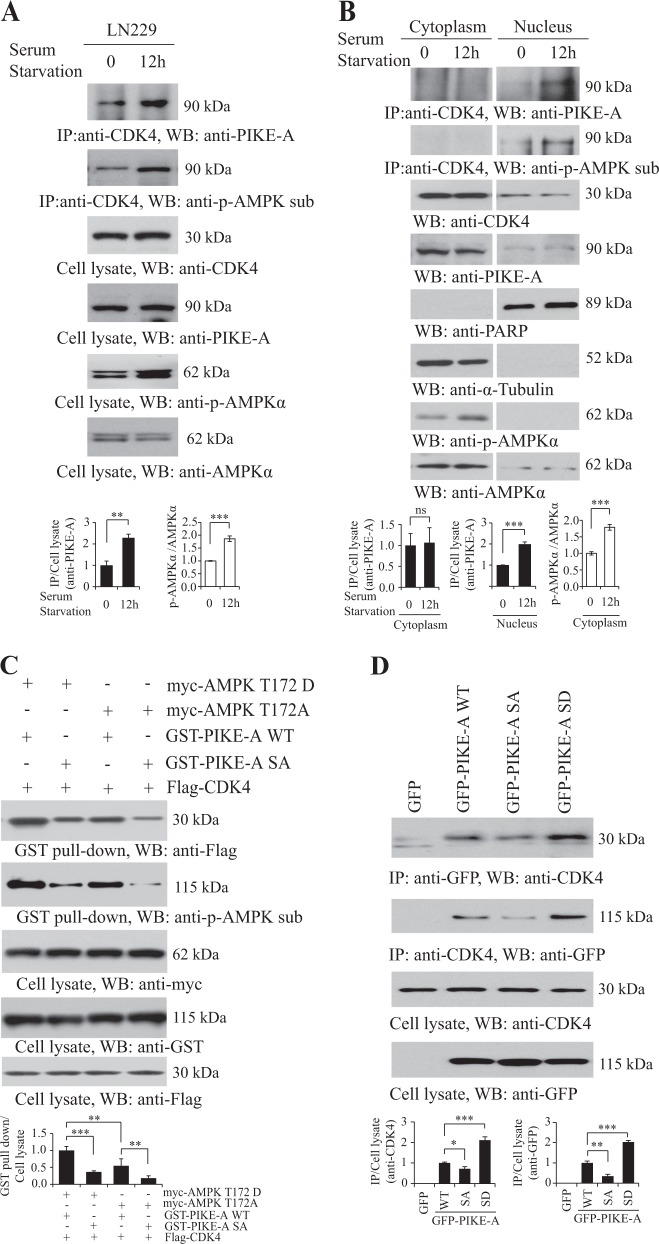
PIKE-A phosphorylation by AMPK stimulates its association with CDK4. **a** Serum starvation enhances the interaction of PIKE-A and CDK4. LN229 cells were serum starved for 12 h, followed by immunoprecipitation with anti-CDK4 antibody and immunoblotting using anti-PIKE-A and anti-phospho-(Ser/Thr) AMPK substrate antibody. Quantification is shown at the bottom. **b** Serum starvation enhances the interaction of PIKE-A and CDK4 in nucleus. LN229 cells were serum starved for 12 h, followed by subcellular fractionation. Then cytosolic and nuclear cell lysates were immunoprecipitated with anti-CDK4 antibody and immunoblotted using anti-PIKE-A and anti-phospho-(Ser/Thr) AMPK substrate antibody. Quantification is shown at the bottom. **c** HEK293 cells were co-transfected with Flag-CDK4 with GST-PIKE WT or phospho-deficient GST-PIKE SA mutant in the presence of myc-AMPKα T172D or myc-AMPKα T172A. PIKE-A was pulled down with glutathione beads and co-precipitated proteins were analyzed by immunoblotting with anti-Flag or anti-phospho-(Ser/Thr) AMPK substrate antibody. The expression levels of transfected constructs were analyzed by immunoblotting. Quantification is shown at the bottom. **d** Different GFP-tagged PIKE-A WT and mutants were transfected into HEK293 cells. Cell lysates were immunoprecipitated with anti-GFP (or anti-CDK4) antibody, and co-precipitated proteins were analyzed by immunoblotting with anti-CDK4 (or anti-GFP) antibody. Quantification is shown at the bottom. All results performed above are presented as mean ± SD from three independent experiments. *p < 0.05; **p < 0.01; ***p < 0.001, ns not significant

Qi’s study showed that PIKE-A directly interacts with CDK4 to form the complex, which promotes cell proliferation and GBM tumorigenesis in vitro and in vivo^21^. Accordingly, we investigated the pathological consequence of PIKE-A nuclear translocation under energy stress, and observed that PIKE-A associated tightly with CDK4 in LN229 cells under serum starvation in the whole cell, particularly in nucleus, but not in cytoplasm (Fig. 4a, b). Similarly, in the presence of AICAR, Metformin, A23187, or H_2_O_2_ stimulation the interaction was enhanced in the nucleus or whole cell, but not in cytoplasm (Figure S4A and S4B). We next measured the interaction between PIKE-A WT or SA mutants and CDK4 when co-transfected with constitutively active (T172D) or inactive (T172A) mutant of AMPKα. The GST-pull down assay displayed that compared with the prominent interaction between PIKE-A WT and CDK4, phospho-deficient PIKE-A mutant SA revealed lower binding affinity to CDK4. AMPKα T172D provoked the PIKE-A WT/CDK4 association, but its stimulatory effect was reduced when PIKE-ASA was employed. As expected, PIKE-A SA barely interacted with CDK4 in the presence of AMPKα T172A (Fig. 4c). Furthermore, when co-transfected CDK4 with PIKE-A WT, SA or SD, the co-immunoprecipitation assay results showed that phospho-mimetic SD mutant of PIKE-A escalated the interaction between CDK4 and PIKE-A, whereas the SA mutant blocked this interaction (Fig. 4d). Hence, these data strongly suggest that AMPK phosphorylation regulates the interaction between PIKE-A and CDK4.

### AMPK phosphorylation of PIKE-A prevents CDK4-Rb signaling pathway

**Fig. 5:**
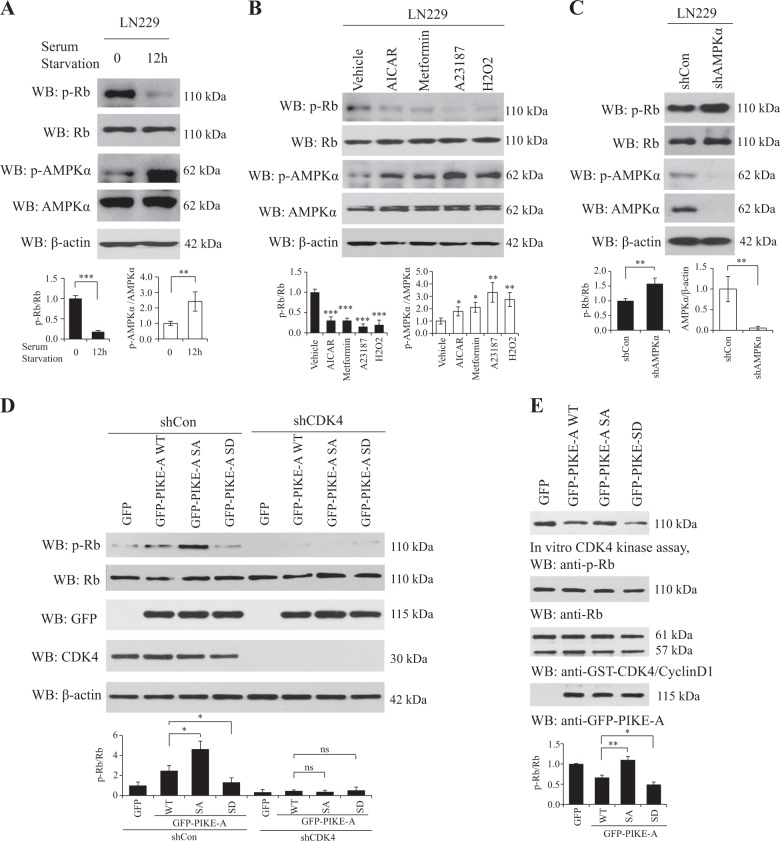
PIKE-A phosphorylation regulates the CyclinD1/CDK4 pathway. **a** Serum starvation decreases Rb phosphorylation. LN229 cells were serum starved for 12 h. Cell lysates were immunoblotted using anti-p-Rb antibody. Quantification is shown at the bottom. **b** AMPK activators inhibit Rb phosphorylation. LN229 cells were treated with AICAR, Metformin, A23187, and H_2_O_2_. Then Rb, as a downstream effector of CDK4, was analyzed by immunoblotting. Quantification is shown at the bottom. **c** Knock down of AMPKα increases Rb phosphorylation. Rb phosphorylation was analyzed by immunoblotting in AMPKα shRNA or control vector-transfected LN229 cells. Quantification is shown at the bottom. **d** PIKE-A phosphorylation suppresses CDK4-mediated Rb phosphorylation. In LN229 cells, CDK4 shRNA or control shRNA were transfected with GFP-PIKE-A WT and mutants (SA and SD). Cell lysates were immunoblotted using anti-p-Rb antibody. Quantification is shown at the bottom. **e** PIKE-A phosphorylation inhibits CDK4 activity in vitro. Purified GFP-PIKE-A WT or mutants (SA and SD) recombinant proteins were incubated with active CyclinD1/CDK4 complex, which were then incubated with Rb peptide in kinase reaction buffer for 30 min at 30 °C. Rb phosphorylation statuses were analyzed by immunoblotting. Quantification is shown at the bottom. All results performed above are presented as mean ± SD from three independent experiments. *p < 0.05; **p < 0.01; ***p < 0.001, ns not significant

To explore the effects of PIKE-A, CDK4, and their combined effect on downstream signaling cascades, we performed Rb phosphorylation analysis under different AMPK activation conditions. Rb is one of the major downstream targets of CDK4, and p-Rb signals are prominently elevated when CDK4 is overexpressed^22^. Our results showed that induction of AMPK activity by serum starvation and the well-characterized stimuli blocked the Rb phosphorylation level (Fig. 5a, b). In contrast, when we knocked down AMPK, Rb phosphorylation was elevated (Fig. 5c). Then we investigated the effect of AMPK on phosphorylation of PIKE-A in mediating CDK4-Rb signaling cascades. Overexpression of PIKE-A WT or SA but not SD strongly elevated Rb phosphorylation, in alignment with what was observed in GFP empty vector-transfected cells, and these effects were abolished when knocked down by CDK4 (Fig. 5d).

To examine whether PIKE-A directly inhibits CDK4 activity, we performed in vitro CDK4 kinase assay employing the Rb peptide as a CDK4 substrate. When purified GFP-PIKE-A WT or mutant (SA and SD) recombinant proteins were incubated with the active CyclinD1/CDK4 complex, immunoblotting assay showed that PIKE-A SD strongly blocked CDK4 activity compared with PIKE-A or SA, suggesting that phosphorylated PIKE-A binds to CDK4 and inhibits its kinase activity (Fig. 5e). Collectively, our data support that PIKE-A phosphorylation suppresses the CDK4-Rb pathway, which is mediated by cellular energy stress-induced AMPK activation.

### AMPK-phosphorylated PIKE-A suppresses cell proliferation in GBM cells

**Fig. 6:**
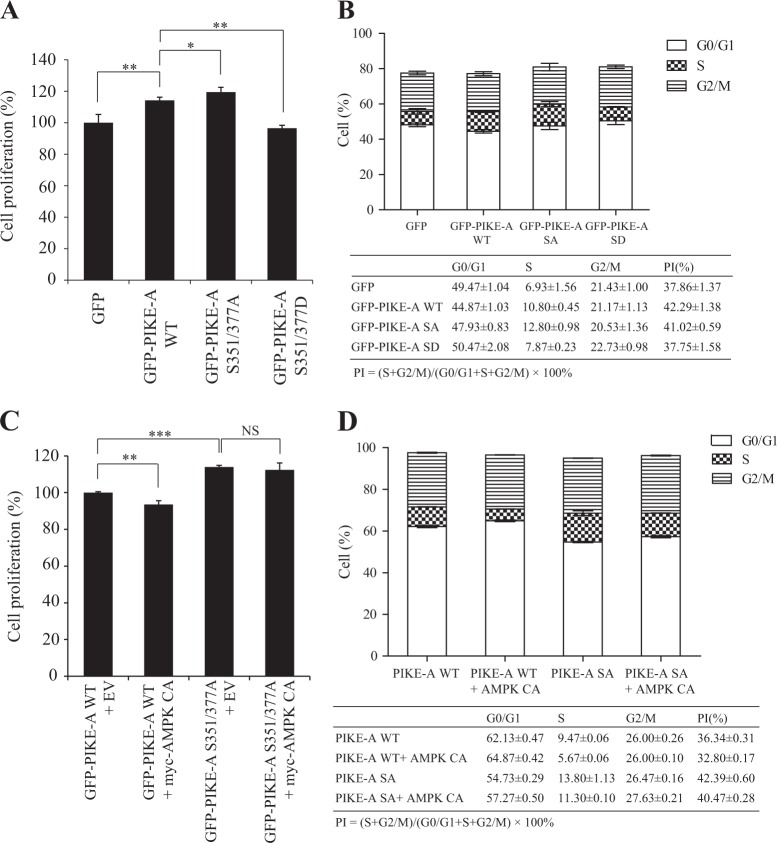
AMPK-phosphorylated PIKE-A suppresses cell proliferation in GBM cells. **a** LN229 cells were transfected with GFP-PIKE-A WT and mutant (SA and SD) and cell proliferation was tested by cell counting. **b** LN229 cells were transfected with GFP-PIKE-A WT and mutant (SA and SD) and cell cycle distributions were analyzed by flow cytometry. Upper: Percentages of cells in phases of G0/G1, S, and G2/M are indicated. Lower: Cell proliferation index (PI) was calculated based on the indicated equation and is shown. **c** LN229 cells were co-transfected with GFP-PIKE-A WT or SA mutant and constitutively active mutant of AMPKα and cell proliferation was determined by cell counting. **d** LN229 cells were co-transfected with GFP-PIKE-A WT or SA mutant and constitutive active mutant of AMPKα. Cell cycle distributions were analyzed by flow cytometry. Upper: Percentages of cells in phases of G0/G1, S, and G2/M were indicated. Lower: Cell proliferation index (PI) was calculated based on the indicated equation and is shown. All results performed above are presented as mean ± SD from three independent experiments. *p < 0.05; **p < 0.01; ***p < 0.001, ns not significant

AMPK directly phosphorylates PIKE-A on S351/377 and affects its translocation into the nucleus. Therefore, we explored whether S351/377 phosphorylation affects PIKE-A biological consequence in glioblastoma cells. We performed cell proliferation, cell viability, and cell cycle assay in LN229 GBM cells transfected with GFP vector, GFP-PIKE-A WT, SA, and SD mutant, respectively. As expected, PIKE-A WT strongly conferred cell proliferation potential. However, PIKE-A SD mutant, which mimics PIKE-A phosphorylation by AMPK, lost the ability to promote cell proliferation and cell viability (Fig. 6a and Fig. S5A). Furthermore, we observed the similar pattern in G0/G1 to S phase transition (Fig. 6b). Next, we monitored the effect of PIKE-A WT or SA mutant on cell proliferation, cell viability, and cell cycle when co-transfected with active AMPK. The results show that active AMPK strongly inhibits PIKE-A WT but not PIKE-A SA mutant cell proliferation, cell viability, and cell cycle (Fig. 6c, d and Fig. S5B). Through the survival analysis in GBM (WHO grade IV), we found that the low levels of AMPKα phosphorylation (p-AMPKα T172) are significantly correlated with a worse prognosis of patients in TCPA (The Cancer Proteome Atlas, https://tcpaportal.org/tcpa/) datasets, which are based on Reverse phase protein array (RPPA) from TCGA data (Fig. S5C). Moreover, higher mRNA levels of PIKE-A (AGAP2) are correlated with unfavorable clinical outcomes based on the publicly available cBioPortal for Cancer Genomics (http://www.cbioportal.org/) (Fig. S5D). Therefore, our findings indicate that AMPK-phosphorylated PIKE-A induces cell cycle arrest and inhibits cell proliferation.

## Discussion

Extensive studies have revealed that the PIKE-A has an essential function in promoting cancer cell survival and growth and preventing cell apoptosis^1–4^. Recent studies have revealed that PIKE-A can be phosphorylated by CDK5, Akt, and Fyn on Ser-279 (ref. ^8^), Ser-629 (ref. ^5^)/Ser-472 (ref. ^7^), and Tyr-682/774 (ref. ^11^), respectively. In addition, it has been shown that PIKE-A can also be regulated by extracellular signals, such as epidermal growth factor (EGF). In this study, we show that intracellular metabolic/energy stress regulates PIKE-A phosphorylation and nuclear translocation mediated by AMPK activation. Therefore, PIKE-A can integrate and coordinate both extracellular and intracellular signals under energy stress.

AMPK detects cellular energy stress, which modulates cellular metabolism balance and limits cell growth^13^. AMPK accomplishes its regulatory functions either via direct and rapid phosphorylation of the metabolic enzymes or eliciting indirectly target gene expression^15,29^. Our present study identifies that AMPK directly phosphorylates PIKE-A in response to cellular energy stress. It is worth noting that the phosphorylation sites of PIKE-A are Ser-351 and Ser-377, which are in the PH domain (Fig. 1). Notably, our subsequent data indicated that these two phosphorylated sites of PIKE-A display the same biological function. Our previous report showed that the GTPase domain of PIKE-A is responsible for binding AMPK^24^. We propose that this binding is conducive and sufficient for PIKE-A phosphorylation by AMPK. Further evidence supports that a physiological function of PIKE-A phosphorylation in cellular energy response is PIKE-A nuclear translocation, which is dependent on AMPK activation (Fig. 2). The mechanisms underlying the nuclear translocation of PIKE-A and its role in tumorigenesis were not previously well understood. We demonstrate here that the nuclear trafficking of PIKE-A is regulated by 14-3-3, which contains a bipartite nuclear localization signal (NLS) and consequently promotes PIKE-A binding to CDK4 tightly in the nucleus (Figs. 3 and 4). This interaction shields CDK4 and disrupts the CDK4–CyclinD1 complex formation and Rb activity, and further induces cell cycle arrest (Figs 5 and 6). Therefore, PIKE-A phosphorylation is likely to play a role in maintaining cellular energy homeostasis. Energy-stress-induced PIKE-A phosphorylation and nuclear translocation mediated by AMPK activation reduces energy expenditure and cell growth, possibly by inhibiting the CDK4–Rb pathway. Our study uncovers a mechanism of cellular energy and crosstalk with PIKE-A through mechanisms of AMPK regulation.

Considering the general role of PIKE-A in promoting cell proliferation and inhibiting apoptosis through PIKE-A modification such as phosphorylation and cleavage, etc., it is not surprising that PIKE-A phosphorylation is coordinated with cellular stress status. When cell conditions are normal or favorable, PIKE-A activates Akt and promotes cell proliferation. However, cell proliferation should not proceed if cellular energy is limited, and such conditions would allow AMPK activation to maintain basic survival. Therefore, the direct phosphorylation of PIKE-A in an AMPK-dependent manner provides a mechanism to ensure that cell proliferation occurs only when favorable growth conditions are available. The phosphorylation of PIKE-A by AMPK adds new dimensions to both energy stress-mediated regulations of PIKE-A and the pathomechanisms of AMPK in controlling of cell growth. Indeed, phosphorylation of PIKE-A has the significant benefit of cell proliferation blockade and provides new target for therapeutic intervention of cancer. Thus, high activation of AMPK may pave the way for improved outcomes for cancer patients with high PIKE-A expression.

## Materials and methods

### Cell culture and reagents

LN229 glioblastoma cells were maintained in Dulbecco's modified Eagle's medium containing 10% fetal bovine serum at 37 °C with 5% CO_2_ atmosphere in a humidified incubator. This cell line was a gift from Dr. Keqiang Ye’s lab and has been previously described^24,26^. AICAR, Metformin hydrochloride, Calcium Ionophore A23187, antibody against GFP, and GST-CDK4/CyclinD1 protein were from Sigma-Aldrich (St. Louis, MO, USA). Antibody against myc, GST, p-AMPK α (T172), AMPK α, p-AMPK α substrate, PARP, α-Tubulin, p-Rb, Rb was from Cell Signaling Technology (Beverly, MA, USA). Antibody against Flag, CDK4, and PIKE-A were purchased from Santa Cruz Biotechnology (Santa Cruz, CA, USA). Control and targeted hairpins against AMPK α (shAMPKα) and 14-3-3β (sh14-3-3β) were purchased from Thermo Fisher Scientific (Waltham, MA, USA).

### In vitro phosphorylation assay

After transfection, 500 μg protein from each sample were prepared and immunoprecipitated by adding 2 μl anti-GFP antibody and 25 μl of protein A-G agarose (Santa Cruz) at 4 °C for 3 h. Phosphorylation reactions were performed with immunoprecipitated GFP-PIKE-A from 500 μg total protein and 0.1 μg of active AMPKα1β1γ1 (SignalChem) in a final volume of 50 μl 10× AMPK kinase buffer (5 mM MOPS, pH 7.2, 2.5 mM β-glycerophosphate, 1 mM EGTA, 0.4 mM EDTA, 5 mM MgCl_2_, 0.05 mM DTT) and 1 μl [γ-32P]ATP (Perkin Elmer). Selected reactions were carried out in the presence or absence of active AMPKα1β1γ1. After incubation at 30 °C for 30 min, the reactions were terminated by addition of 2.5 μl of 5× sodium dodecyl sulfate (SDS) buffer, and the samples were subjected to 12.5% SDS-polyacrylamide gel electrophoresis (PAGE). The gels were dried with a Model 583 Gel Dryer (Bio-Rad) and phosphorylated proteins were visualized by autoradiography.

### Cytoplasmic and nuclear fractionation

LN229 cells were collected and wash once with ice-cold 1× phosphate-buffered saline (PBS). The cell pellet was resuspended in CER I buffer. The cytoplasmic and nuclear fractions were prepared as described in the manufacturer’s protocol (PIERCE, Rockford, IL, USA, NE-PER, nuclear and cytoplasmic extraction reagent).

### Co-immunoprecipitation and in vitro-binding assays

These methods were performed essentially as described previously^30^.

### Cell proliferation and cell viability assay

5 × 10^4^ cells were seeded in a six-well plate and cultured at 37 °C for 3 days. Cell proliferation was determined by recording cell numbers 1, 2, and 3 days post-seeding, and normalizing to cell numbers at 0 day. 5 × 10^3^ cells were seeded in a 96-well plate 24 h before the assay starts and were cultured at 37 °C for 3 days. Cell viability was determined by using CellTiter 96® AQueous One Solution Cell Proliferation Assay (MTS) (Promega).

### Cell cycle assay

LN229 WT, SA, and SD rescue cells (1 × 10^6^ cells) were harvested by trypsinization and washed twice with PBS. After centrifugation, the cells were resuspended in 5 ml of 70% ethanol at 4 °C for 4 h. After rinsing with PBS, the fixed cells were resuspended in PBS containing 50 μg/ml RNaseA and 50 μg/ml propidium iodide and incubated at 4 °C for 4 h. The stained cells were passed through a nylon-mesh sieve to remove cell clumps and were analyzed by a FACScan flow cytometer.

### In vitro CDK4 kinase assay

GFP-PIKE-A WT, SA, or SD was transfected into HEK293 cells, and then 1 mg protein from each sample was prepared and immunoprecipitated by adding 2 μl anti-GFP antibody and 25 μl of protein A-G agarose (Santa Cruz) at 4 °C for 3 h. CDK4 kinase analysis was performed with the GST-tagged recombinant Active CDK4/Cyclin D1 complex (Sigma-Aldrich), recombinant amino acids 769–921 mapping within the carboxy-terminal domain of Rb (Santa Cruz), GFP-PIKE-A WT, SA, or SD extract with 10 mM ATP and 25 mM MOPS (pH 7.2), 12.5 mM β-glycerolphosphate, 25 mM MgCl_2_, 5 mM EGTA (pH 8.0), 2 mM EDTA (pH 8.0), and 0.25 mM DTT, and incubated at 30 °C for 30 min. The reactions were stopped by addition of sample buffer containing 125 mM Tris-HCl (pH 6.8), 10% β-mercaptoethanol, 9.2% SDS, 0.04% bromphenol blue, and 20% glycerol and boiled for 5 min. Samples were resolved by SDS-PAGE, and phosphorylation of Rb was measured by WB analysis.

### In silico study

The Reverse phase protein array (RPPA) data on TCGA-GBM patients were obtained from TCPA (https://tcpaportal.org/tcpa/TCGA-GBM-L4.zip). The survival data of these 204 GBM patients were downloaded from TCGA (https://portal.gdc.cancer.gov/). Data were analyzed by log-rank test and KM plots were drawn by Graphpad Prism 7.0.

### Statistical analysis

Data are shown as mean ± SD from three independent experiments. The *p* values of less than 0.05 were considered statistically significant, *p < 0.05. Statistical differences were calculated with unpaired two-tailed Student’s *t*-test using GraghPad prism software.

## Supplementary information

Supplementary information

SUPPLEMENTAL Figure 1

SUPPLEMENTAL Figure 2

SUPPLEMENTAL Figure 3

SUPPLEMENTAL Figure 4

SUPPLEMENTAL Figure 5
