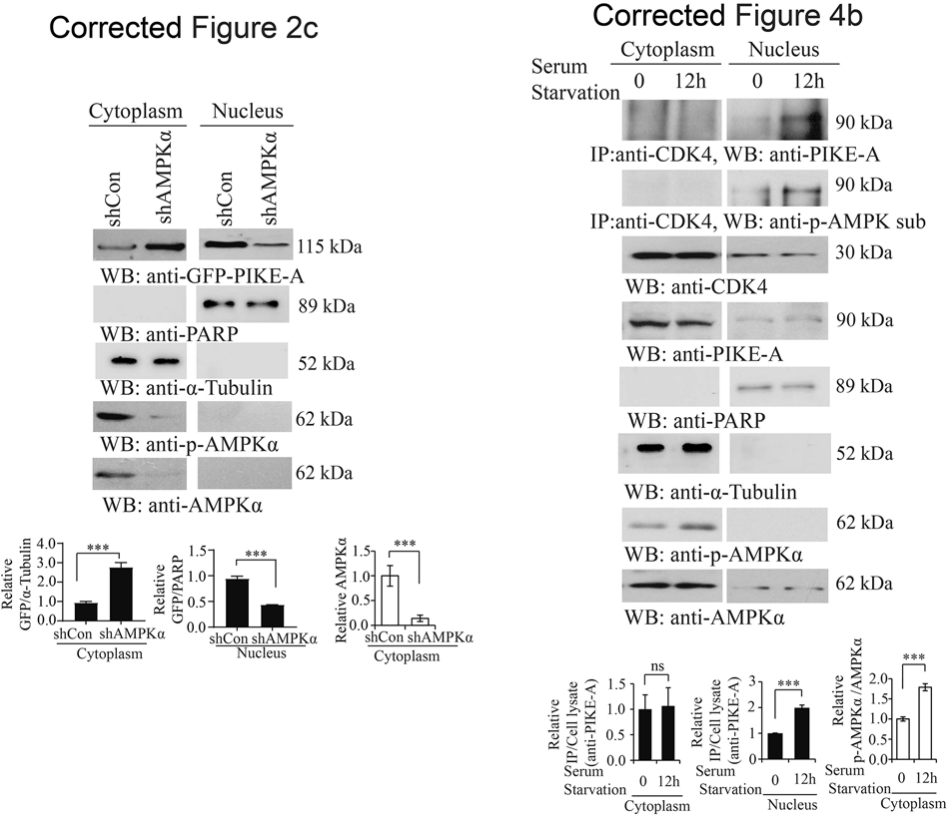# Correction to: Cellular energy stress induces AMPK-mediated regulation of glioblastoma cell proliferation by PIKE-A phosphorylation

**DOI:** 10.1038/s41419-025-07700-2

**Published:** 2025-06-12

**Authors:** Shuai Zhang, Hao Sheng, Xiaoya Zhang, Qi Qi, Chi Bun Chan, Leilei Li, Changliang Shan, Keqiang Ye

**Affiliations:** 1 Department of Medical Biochemistry and Molecular Biology, School of Medicine, Jinan University, 510632 Guangzhou, Guangdong China; 2 Department of Pathology and Laboratory Medicine, Emory University School of Medicine, Atlanta, GA 30322 USA; 3 The First Affiliated Hospital, Biomedical Translational Research Institute, Jinan University, 510632 Guangzhou, Guangdong China; 4 Department of Pharmacology and Emory Chemical Biology Discovery Center, Emory University, Atlanta, GA 30322 USA; 5 Department of Pharmacology, School of Medicine, Jinan University, 510632 Guangzhou, Guangdong China; 6 School of Biological Sciences, The University of Hong Kong, Hong Kong SAR, China

Correction to: *Cell Death and* *Disease* 10.1038/s41419-019-1452-1, published online 4 March 2019

We found two errors in the graph of the article. The blank, anti-PARP, and anti-α-Tubulin of Figs. 2c and 4b were wrongly used during the figure assembly process. Now, the error has now been corrected. The updated Figs. 2c and 4b with the correct images are provided in this correction. This error does not affect the conclusions of the study.

**Originally published Figs. 2c and 4b** 
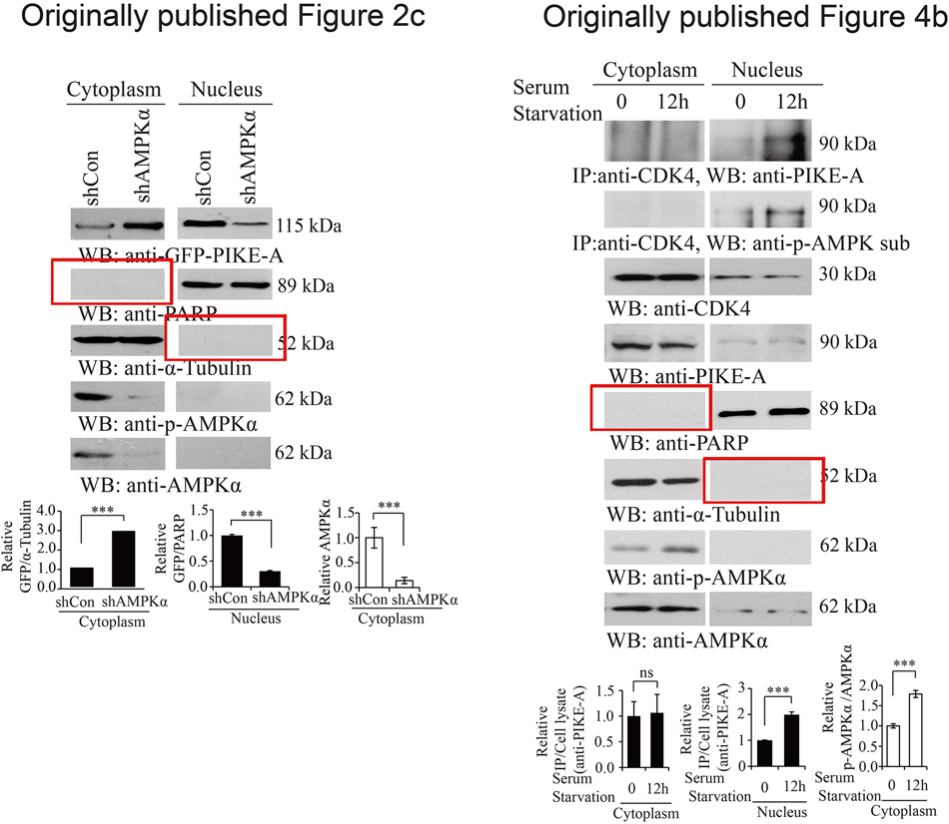


**Corrected Figs. 2c and 4b**