# Supplementary material for: Cellular energy stress induces AMPK-mediated regulation of glioblastoma cell proliferation by PIKE-A phosphorylation
Source: Cell Death Dis. 2019 Mar 4;10(3):222. doi: 10.1038/s41419-019-1452-1 (PMC6399291; doi:10.1038/s41419-019-1452-1)
Supplement: Supplementary file 1 — Supplementary information [file 41419_2019_1452_MOESM1_ESM.docx]

**Cellular energy stress induces AMPK-mediated regulation of glioblastoma cell proliferation by PIKE-A phosphorylation**

*Running title: AMPK phosphorylates PIKE-A under energy stress*

Shuai Zhang^1,2^, Hao Sheng^3^, Xiaoya Zhang^3^,Qi Qi^4,5^, Chi Bun Chan^6^, Leilei Li^3^, Changliang Shan^3*^and Keqiang Ye^2*^

^1^ Department of Medical Biochemistry and Molecular Biology, School of Medicine, Jinan University, Guangzhou, 510632 Guangdong, China;

^2^ Department of Pathology and Laboratory Medicine, Emory University School of Medicine, Atlanta, 30322 GA, USA;

^3^The First Affiliated Hospital, Biomedical Translational Research Institute, Jinan University, Guangzhou, 510632 Guangdong, China;

^4^Department of Pharmacology and Emory Chemical Biology Discovery Center, Emory University, Atlanta, 30322 GA, USA;

^5^Department of Pharmacology, School of Medicine, Jinan University, Guangzhou, 510632 Guangdong, China;

^6^School of Biological Sciences, The University of Hong Kong, Hong Kong SAR, China

**^*^Correspondence Authors:** C Shan, The First Affiliated Hospital, Biomedical Translational Research Institute, Jinan University, Room 808, Liang Zhongjing Building, 601 Huangpu Avenue West, Guangzhou, Guangdong 510632, China. Tel: +86-2085222787; Fax: +86-2085222787; E-mail: [changliangshan@jnu.edu](mailto:jchen@emory.edu).cn(C.S.); K Ye, Department of Pathology and Laboratory Medicine, Emory University School of Medicine, Room 141, Whitehead Building, 615 Michael Street, Atlanta, 30322 GA, USA. Tel: +404 712 2814; Fax: +404 712 2979; E-mail: [kye@emory.edu](mailto:kye@emory.edu)

**Supplementary Figure Legends**

**Figure S1. AMPK phosphorylates PIKE-A on serine 351 and 377 residues.** (A) PH domain of PIKE-A is phosphorylated by AMPK. A series of myc-tagged PIKE-A truncations were incubated with active AMPK (α1β1γ1) and detected by autoradiography. (B) AMPK phosphorylates PIKE-A on serine 351 and 377 residues. Immunoprecipitated myc-tagged PIKE-A WT and mutants (S351A, S377A and SA) were detected by anti- phospho-(Ser/Thr) AMPK substrate antibody.

**Figure S2 AMPK phosphorylates PIKE-A and stimulates its nuclear translocation.** (A-B) Serum starvation or hypoxia induces PIKE-A phosphorylation. LN229 cells were serum starved (A) or subjected to hypoxia (B) for 12h. Endogenous PIKE-A was then precipitated by anti-PIKE-A antibody and its phosphorylation level was detected by anti- phospho-(Ser/Thr) AMPK substrate antibody. (C) AMPK activator enhances PIKE-A nuclear localization. LN229 cells were transfected with GFP-PIKE-A WT and then treated with AICAR, Metformin, A23187 and H_2_O_2_, followed by subcellular fractionation. The purity of the cytosolic and nuclear fractions was confirmed by the absence of α-tubulin in the nuclear fraction and PARP in the cytosolic fraction.

**Figure S3. AMPK activator increases the interaction of PIKE-A and 14-3-3β.** HEK293 cells were co-transfected with GST-PIKE-A and GFP-14-3-3β and then treated with AICAR, Metformin, A23187 and H_2_O_2_, followed by immunoprecipitation.

**Figure S4. AMPK activator increases the interaction of PIKE-A and CDK4**. (A) LN229 cells were treated with AICAR, Metformin, A23187 and H2O2. Cell lysate was immunoprecipitated with anti-CDK4 antibody and immunoblotted using anti-PIKE-A and anti-phospho-(Ser/Thr) AMPK substrate antibody. (B) LN229 cells were treated with AICAR, Metformin, A23187 and H_2_O_2_, followed by subcellular fractionation. Cytosolic and nuclear cell lysates were immunoprecipitated with anti-CDK4 antibody and immunoblotted using anti-PIKE-A and anti- phospho-(Ser/Thr) AMPK substrate antibody.

**Figure S5. AMPK-phosphorylated PIKE-A suppresses cell proliferation in GBM cells.** (A) LN229 cells were transfected with GFP-PIKE-A WT and mutant (SA and SD) and cell viability were tested by MTS assay. (B) LN229 cells were co-transfected with GFP-PIKE-A WT or SA mutant and constitutive active mutant of AMPKα and cell viability were tested by MTS assay. (C) Kaplan–Meier survival curves for correlation between the low levels of AMPKα phosphorylation (p-AMPKα T172) and survival of GBM patients in the TCPA dataset. This data were retrieved at TCGA-GBM-L4.zip from TCPA. Significance was determined by the log-rank test(*p ≤ 0.05). (D) Kaplan–Meier curves of overall survival in GBM patients with mRNA level of PIKE-A (AGAP2), calculated from [cBioPortal for Cancer Genomics](http://www.baidu.com/link?url=QQw6MmcxNx3zzkHgb9JxDR1c0DD_3MnVesQ0r3d0WLWyyJYAhBHocZsyNRrTzm63) (<http://www.cbioportal.org/>). Significance was determined by the log-rank test(*p ≤ 0.05).
