# Supplementary figures and images for: Cellular energy stress induces AMPK-mediated regulation of glioblastoma cell proliferation by PIKE-A phosphorylation
Source: Cell Death Dis. 2019 Mar 4;10(3):222. doi: 10.1038/s41419-019-1452-1 (PMC6399291; doi:10.1038/s41419-019-1452-1)

Figure S1

A

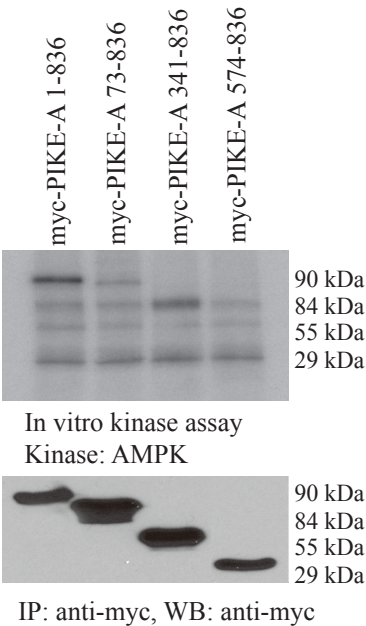

B

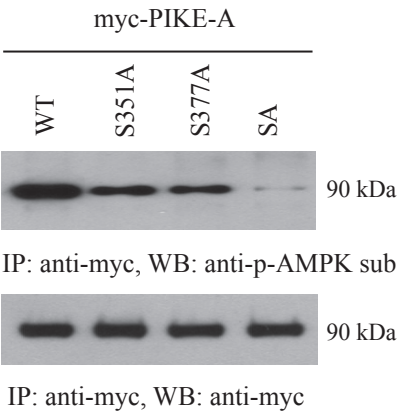

Supplement: Supplementary file 2 — SUPPLEMENTAL Figure 1 [file 41419_2019_1452_MOESM2_ESM.pdf]

Figure S2

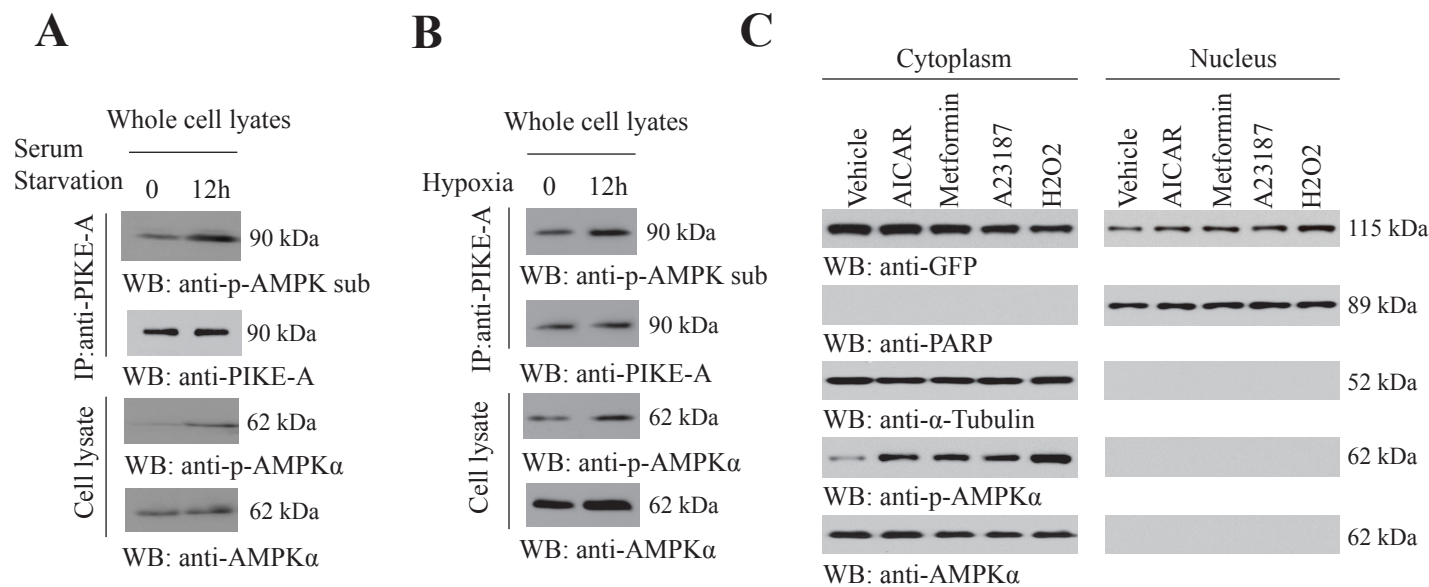

Supplement: Supplementary file 3 — SUPPLEMENTAL Figure 2 [file 41419_2019_1452_MOESM3_ESM.pdf]

## Figure S3

A

GST-PIKE-A WT / GFP-14-3-3 $\beta$

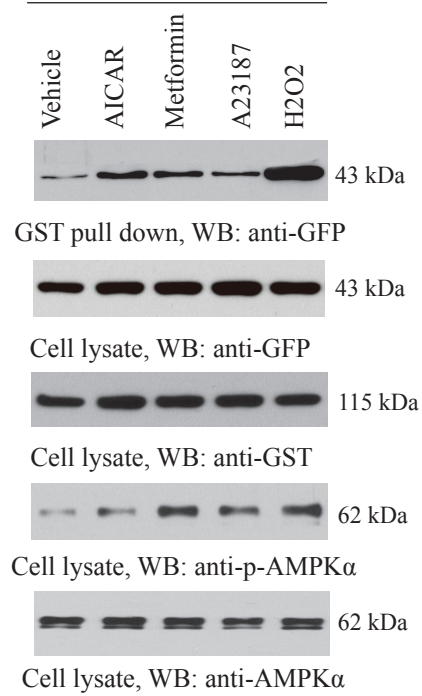

Supplement: Supplementary file 4 — SUPPLEMENTAL Figure 3 [file 41419_2019_1452_MOESM4_ESM.pdf]

Figure S4

A

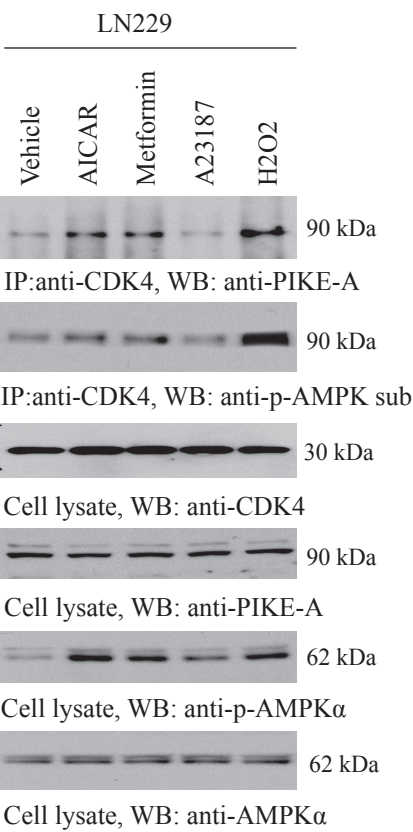

B

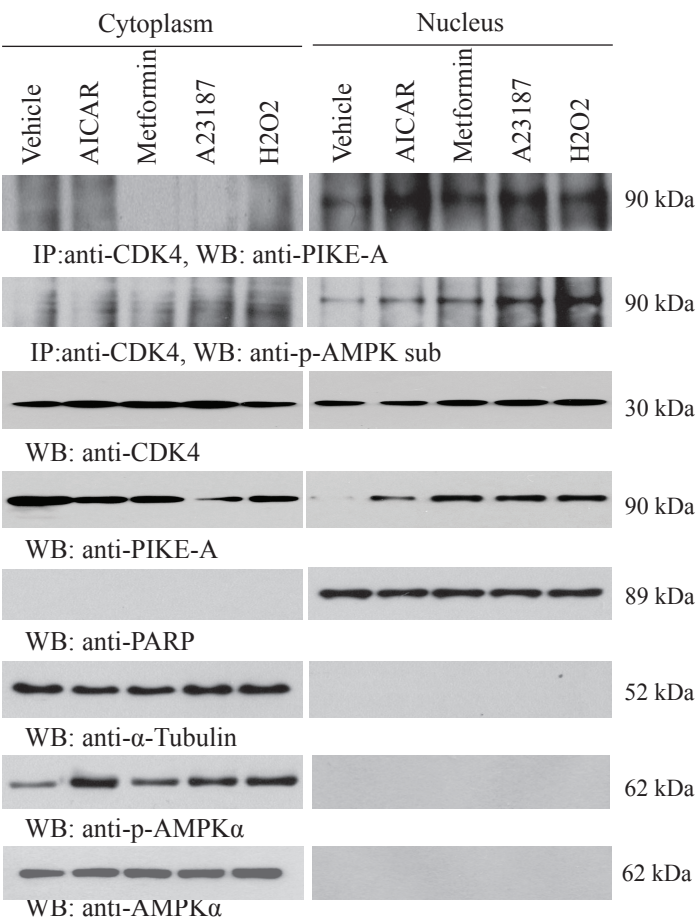

Supplement: Supplementary file 5 — SUPPLEMENTAL Figure 4 [file 41419_2019_1452_MOESM5_ESM.pdf]

**Figure S5**

**A**

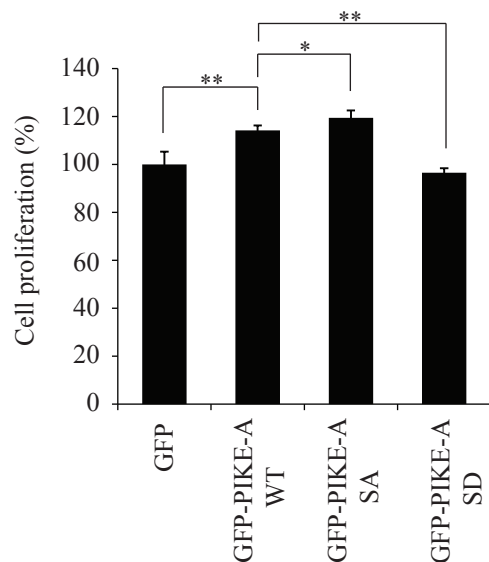

**B**

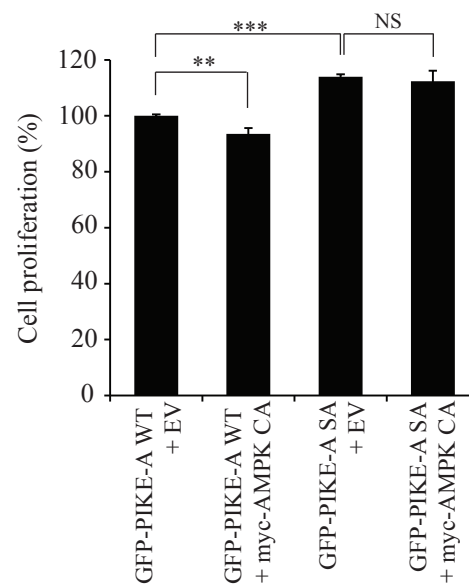

**C**

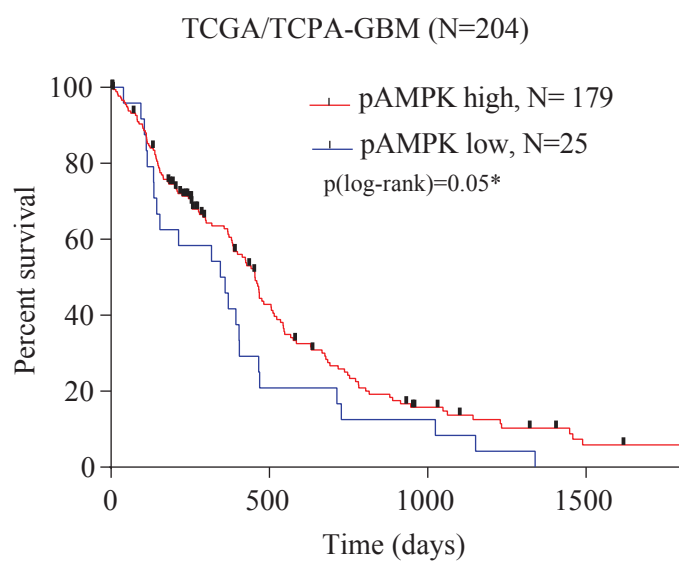

**D**

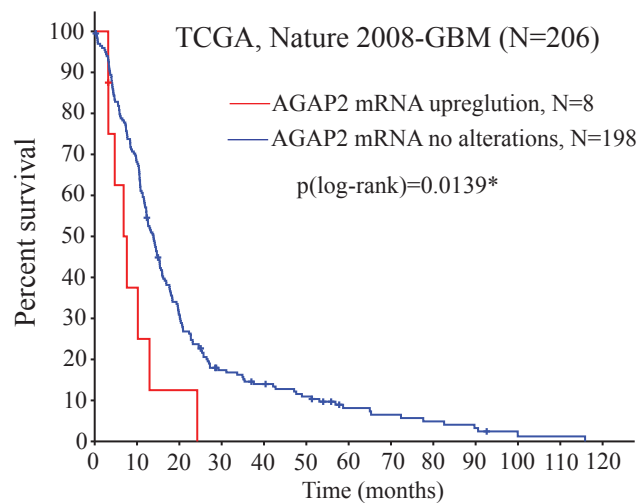

Supplement: Supplementary file 6 — SUPPLEMENTAL Figure 5 [file 41419_2019_1452_MOESM6_ESM.pdf]
